# Successful Management of Total Intravenous Anesthesia Using Remimazolam, Dexmedetomidine, and Remifentanil for Airway Stenting Under Rigid Bronchoscopy: A Case Report

**DOI:** 10.1002/ccr3.73032

**Published:** 2026-06-25

**Authors:** Yoko Kurihara, Maiko Hosokawa, Kiyoko Bito, Yusuke Ishida, Mariko Saito, Rena Fukura, Katsunori Oe

**Affiliations:** ^1^ Department of Anesthesiology Showa Medical University School of Medicine Shinagawa‐ku Tokyo Japan

**Keywords:** airway stenting, dexmedetomidine, high flow nasal cannula oxygen, remimazolam, rigid bronchoscope

## Abstract

During airway stenting for airway stenosis under rigid bronchoscopy, preservation of spontaneous breathing is desirable. We describe safe anesthesia management for left bronchial stent insertion for cancer‐induced airway stenosis using remifentanil and remimazolam, due to their short half‐life and availability of an antagonist, with maintenance of spontaneous respiration without hypoxemia.

## Graphical abstract

Using intravenous general anesthesia with remimazolam and remifentanil, we successfully performed airway stent placement for cancer‐related airway stenosis without causing hypoxia.

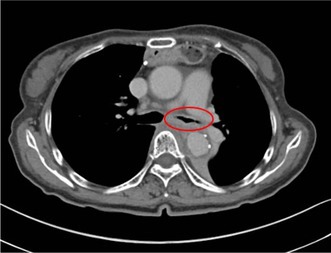

**Abbreviations**
CT: computed tomography
HFNC: high‐flow nasal cannula
PSI: Patient State Index
SpO_2_
: peripheral blood oxygen saturation
VA‐ECMO: veno‐arterial extracorporeal membrane oxygenation
VV‐ECMO: veno‐venous extracorporeal membrane oxygenation

## 1 Introduction

Airway stent placement is performed as a treatment for central airway stenosis or obstruction caused by progression of esophageal or tracheal cancer. Bronchial stent placement for airway stenosis involves the use of a rigid bronchoscope under anesthesia management that preserves spontaneous respiration. However, the anesthesia management is extremely challenging due to concerns such as respiratory depression due to anesthetic agents and the need for prevention of bucking induced by manipulation of the rigid bronchoscope. In particular, overdose of anesthetic agents can easily lead to respiratory arrest, making careful selection and dose adjustment of the anesthetic drugs essential. Here, we present a case in which airway stent placement using a rigid bronchoscope was successfully managed, while preserving spontaneous respiration under general anesthesia with remifentanil, remimazolam, and dexmedetomidine, in combination with high‐flow nasal oxygen therapy. Written, informed consent was obtained from the patient's family for publication of this case report and the accompanying images.

## 2 Case History/Examination

The patient was a woman in her 70s, weighing 48 kg and 157 cm tall, with a medical history of hypertension and atrial fibrillation. She had previously undergone video‐assisted thoracic surgery‐esophagectomy for esophageal cancer 1 year earlier. During follow‐up, she was found to have recurrence of the esophageal cancer, for which she underwent chemotherapy. Thereafter, a contrast‐enhanced chest computed tomography (CT) scan performed for chemotherapy response evaluation revealed stenosis of the left main bronchus due to tumor involvement. Since she also experienced progressively worsening back pain and cough, airway stent placement was planned to alleviate her symptoms. On admission, her physical status was classified as NYHA II and Hugh‐Jones grade III, with an SpO_2_ of 96% on room air. Arterial blood gas analysis showed a PaCO_2_ of 37.7 mmHg and a PaO_2_ of 67.5 mmHg, indicating no hypoxemia or hypercapnia. Contrast‐enhanced CT also revealed a mass at the tracheal bifurcation, stenosis of the left main bronchus, and atelectasis of the left lower lung lobe (Figure 1). Although the preoperative PaO_2_ was 67.5 mmHg and initially interpreted as within an acceptable range, it may indicate mild hypoxemia when adjusted for the patient's age. Furthermore, the presence of left lower lobe atelectasis on CT suggests impaired gas exchange due to intrapulmonary shunt, which may have reduced the patient's respiratory reserve. Given these findings, the patient was considered to have limited pulmonary reserve, which increased the risk of hypoxemia during airway manipulation and sedation.

**FIGURE 1:**
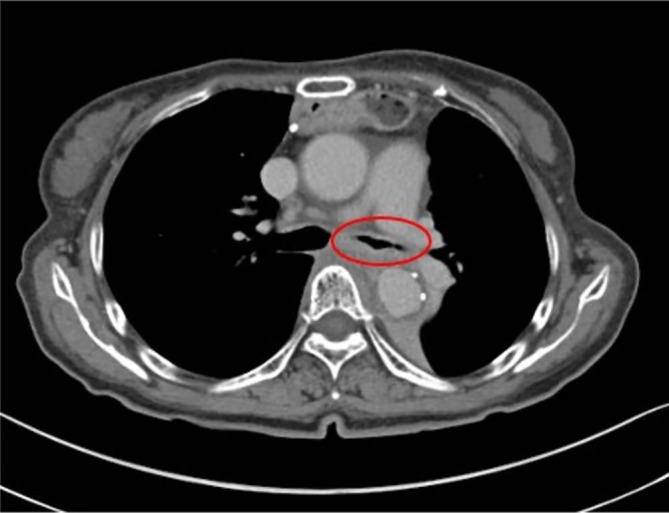
Contrast‐enhanced CT scan. A mass was present at the tracheal bifurcation, and the left main bronchus was found to be narrowed. (red circle).

## 3 Methods (Differential Diagnosis, Investigations, and Treatment)

Bronchoscopy demonstrated extrinsic compression of the left main bronchus.

Since the anesthetic management of bronchial stent placement involves the potential risk of ventilatory difficulty due to loss of spontaneous respiration after anesthetic administration, as well as the risk of tracheal injury if the patient moves during rigid bronchoscope manipulation, we decided on the following anesthetic management strategy: (1) we prioritized the use of sedatives, analgesics, and muscle relaxants with available antagonists to enable prompt intervention in case of hypoxemia due to respiratory arrest or ventilation difficulty. The dosage of these drugs was carefully considered. During intraoperative drug titration, the dexmedetomidine infusion rate was kept constant, the remifentanil dose was not adjusted unless the patient moved, and the remimazolam dose was modified based on the Patient State Index (PSI). Additionally, (2) high‐flow nasal oxygen therapy was administered during rigid bronchoscope insertion to improve oxygenation and facilitate carbon dioxide washout. Furthermore, (3) veno‐venous extracorporeal membrane oxygenation (VV‐ECMO) was prepared as a standby measure in case of unexpected ventilatory difficulty due to intraoperative airway bleeding.

For anesthesia induction, the patient was sufficiently oxygenated with 6 L/min of oxygen before initiating a continuous infusion of remifentanil at 0.1 μg/kg/min, remimazolam at 2 mg/kg/h, and dexmedetomidine at 0.7 μg/kg/h. After confirming loss of consciousness and the ability to perform manual ventilation, 20 mg of rocuronium was administered, followed by tracheal intubation. After induction and tracheal intubation with rocuronium, spontaneous respiration was temporarily suppressed. As the neuromuscular blockade gradually wore off, spontaneous breathing resumed and was confirmed based on thoracic movement and ventilatory patterns. Subsequent anesthetic management was primarily based on the presence of spontaneous respiration.

Additional doses of rocuronium were administered intermittently and only during periods of intense airway stimulation to suppress airway reflexes during rigid bronchoscope manipulation. However, efforts were made to minimize the duration of neuromuscular blockade and allow recovery of spontaneous respiration whenever feasible. To prepare for the risk of intraoperative ventilation difficulty, ECMO access sheaths were placed in both femoral veins under local anesthesia. For anesthesia maintenance, remifentanil was continuously infused at 0.1 μg/kg/min. Dexmedetomidine was initially infused at 0.7 μg/kg/h, although the dose was reduced to 0.4 μg/kg/h due to bradycardia, which was considered a side effect. Remimazolam was continuously infused at 0.4–0.5 mg/kg/h, adjusted based on hemodynamics and the PSI. When the PSI increased, an additional 2 mg of remimazolam was intermittently administered to maintain an appropriate depth of sedation. Invasive arterial blood pressure, SpO_2_, and PSI were monitored intraoperatively (Figure 2). At the start of flexible bronchoscopy, since spontaneous respiration was observed, 10 mg of rocuronium was administered to control reflex responses to stimulation while evaluating the trachea. Next, the tracheal tube was removed, and high‐flow nasal cannula (HFNC) therapy using 100% oxygen at 60 L/min was initiated. Then, a rigid bronchoscope was inserted and the procedure was commenced. During rigid bronchoscopy under HFNC, reliable end‐tidal CO_2_ monitoring was technically difficult. Therefore, ventilation status was assessed based on thoracic movement, respiratory pattern, SpO_2_, and intermittent arterial blood gas analysis during the procedure. An additional 20 mg of rocuronium was administered when the rigid bronchoscope passed through the glottis, to suppress reflex responses to stimulation. Muscle relaxant reversal was planned after the rigid bronchoscope had passed through the glottis. However, based on neuromuscular monitoring and observation of movement of the thorax, it was determined that muscle relaxant reversal was unnecessary, and management continued accordingly. No airway reflexes were observed during the procedure, and the remimazolam dose was adjusted as needed based on PSI. During the procedure, SpO_2_ remained in the 90% range under spontaneous respiration. The anesthesia duration was 3 h and 58 min, while the surgical time was 2 h and 35 min. After the surgery, the surgeon instructed that tracheal intubation and sedation be continued to prevent stent displacement. Hence, a 7‐mm single‐lumen endotracheal tube was reinserted following removal of the rigid bronchoscope, and mechanical ventilation was continued in the intensive care unit under dexmedetomidine sedation. Postoperative evaluation indicated no stent displacement or postoperative airway bleeding. The patient was extubated on the day after surgery and was transferred from the intensive care unit to a general ward on postoperative day 2. During the postoperative rounds, the patient was questioned about intraoperative awareness, but indicated no recall of the surgery.

**FIGURE 2:**
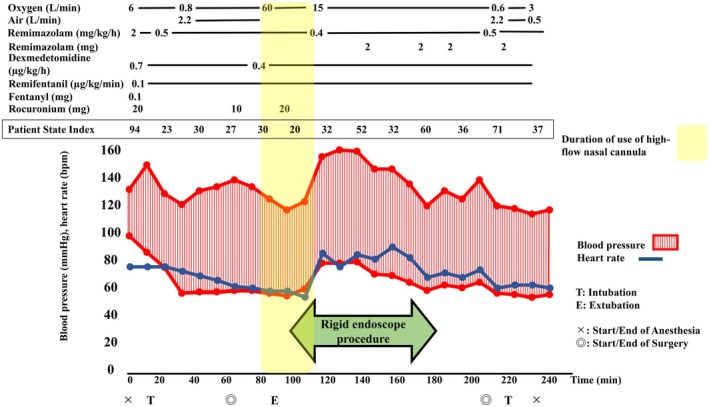
Patient's anesthesia record. Changes in hemodynamic parameters, including heart rate, blood pressure, and Patient State Index, are shown over time. The administration of remimazolam, dexmedetomidine, and remifentanil, as well as the initiation of high‐flow nasal cannula and insertion of the rigid bronchoscope, are indicated.

## 4 Conclusion and Results

We provided safe anesthesia management while preserving spontaneous respiration using intravenous anesthesia with remifentanil, remimazolam, and dexmedetomidine in a patient requiring bronchial stent placement using a rigid bronchoscope.

## 5 Discussion

There are several methods for respiratory management during bronchial stent placement [1]. When performing surgery under spontaneous respiration, sedation and analgesia must be administered while preserving spontaneous breathing, which requires careful selection and precise dosing of anesthetic agents. In this case, remimazolam was used to allow for its rapid reversal in the event of ventilation difficulty during anesthesia induction. Remimazolam is a short‐acting benzodiazepine that became available in Japan in 2020. It has a shorter half‐life than midazolam and can be reversed with flumazenil [2]. A study by Dong et al. reported that remimazolam has a lower incidence of respiratory depression compared to propofol [3]. This suggests that remimazolam might be a safe and effective option for sedation. Additionally, compared to intravenous anesthetics such as propofol and inhalation anesthetics, remimazolam causes less circulatory depression [4, 5]. Thus, remimazolam has several advantages over other sedatives and is used safely across a wide age range. Recent reports have indicated that remimazolam can be safely used not only in adults but also in pediatric patients [6]. However, since it is a relatively new drug, caution is required regarding its potential side effects. To date, serious complications associated with remimazolam include reports of anaphylaxis attributed to the drug and cases of tolerance in patients on long‐term benzodiazepine use [7, 8]. Therefore, caution is necessary when administering remimazolam to such patients.

Dexmedetomidine is an α_2_‐adrenergic agonist that exerts a sedative effect by acting on the locus coeruleus in the brainstem and provides analgesic effects by acting on the dorsal horn of the spinal cord. Additionally, dexmedetomidine has minimal respiratory depressant effects even at high doses [9]. Furthermore, previous reports have indicated that the combination of dexmedetomidine and remimazolam exhibits a favorable synergistic effect [10]. Ribeiro et al. reported that remimazolam is associated with a reduced incidence of respiratory depression and demonstrates improved safety, faster recovery, and high efficacy compared with propofol, dexmedetomidine, and midazolam, supporting its potential as an effective alternative for sedation during bronchoscopy [11]. Because intermittent neuromuscular blockade was required during airway manipulation, careful attention was paid to the risk of intraoperative awareness. In the present case, remimazolam dosing was adjusted according to PSI values and clinical findings to maintain an appropriate depth of anesthesia while avoiding excessive respiratory depression.

Procedures requiring intratracheal manipulation, such as airway stent placement, carry a high risk of airway obstruction due to bleeding, and in some cases, they are performed under ECMO support. However, there are no clear criteria for the indication of ECMO in securing the airway during surgery [12]. Regarding the choice between VV‐ECMO and veno‐arterial (VA)‐ECMO, VA‐ECMO is preferable in cases with concomitant circulatory failure. However, in this case, since there were no circulatory issues and the primary concern was the risk of hypoxemia due to ventilatory failure, we opted to have VV‐ECMO on standby. Fortunately, the procedure was completed in the present case without the need for use of ECMO. The present case is clinically notable because it demonstrates a practical anesthetic strategy for rigid bronchoscopy in a patient with limited pulmonary reserve, combining remimazolam, dexmedetomidine, remifentanil, HFNC, and standby VV‐ECMO while preserving spontaneous respiration whenever feasible.

## Author Contributions

**Yoko Kurihara:** writing – original draft. **Maiko Hosokawa:** writing – original draft, writing – review and editing. **Kiyoko Bito:** writing – review and editing. **Yusuke Ishida:** writing – review and editing. **Mariko Saito:** writing – review and editing. **Rena Fukura:** writing – review and editing. **Katsunori Oe:** writing – review and editing.

## Funding

The authors have nothing to report.

## Ethics Statement

The authors have nothing to report.

## Consent

Written, informed consent was obtained from the patient's family for publication of this case report and the accompanying images.

## Conflicts of Interest

The authors declare no conflicts of interest.

## Data Availability

Data available on request due to privacy/ethical restrictions. The data that support the findings of this study are available on request from the corresponding author. The data are not publicly available due to privacy or ethical restrictions.
